# Multi-octave metasurface-based refractory superabsorber enhanced by a tapered unit-cell structure

**DOI:** 10.1038/s41598-022-21740-0

**Published:** 2022-10-12

**Authors:** Mojtaba Karimi Habil, Maryam Ghahremani, Carlos J. Zapata–Rodríguez

**Affiliations:** 1 Faculty of Physics, University of Tabriz, Tabriz, Iran; 2 Photonics Research Laboratory, Center of Excellence for Applied Electromagnetic Systems, University of Tehran, North Kargar Ave., Tehran, Iran; 3 Department of Optics and Optometry and Vision Sciences, University of Valencia, Dr. Moliner 50, 46100 Burjassot, Spain

**Keywords:** Solar energy and photovoltaic technology, Nanophotonics and plasmonics

## Abstract

An ultra-broadband metasurface-based perfect absorber is proposed based on a periodic array of truncated cone-shaped $\text {TiO}_2$ surrounded by TiN/$\text {TiO}_2$ conical rings. Due to the refractory materials involved in the metasurface, the given structure can keep its structural stability at high temperatures. The proposed structure can achieve a broadband spectrum of 4.3 µm at normal incidence spanning in the range of 0.2–4.5 µm with the absorption higher than $90\%$ and the average absorption around $94.71\%$. The absorption can be tuned through the angle of the cone. By optimizing geometrical parameters, a super absorption is triggered in the range of 0.2–3.25 µm with the absorption higher than 97.40$\%$ and substantially average absorption over 99$\%$. In this regard, the proposed structure can gather more than $99\%$ of the full spectrum of solar radiation. Furthermore, the absorption of the designed structure is almost insensitive to the launching angle up to $50^\circ$ for TE polarization, while it has a weak dependence on the incident angle for TM polarization. The proposed structure can be a promising candidate for thermal energy harvesting and solar absorption applications.

## Introduction

The interaction of the light with plasmonic and high-index materials, which can confine the electromagnetic energy in a small space, has been explored in a variety of optical elements, light trapping applications, and thermal emission control in the infrared region^1–7^. Therefore, an enormous number of studies has been directed to utilize this trapping effect in photonic devices. Among these, electromagnetic wave absorbers are of critical importance having diverse applications in energy harvesting, emitters, light modulation, and sensing. Depending on the absorption bandwidth, absorbers are classified into narrowband and broadband absorbers. The former is notably of use to design chemical and refractive sensors, detectors, and multiband absorbers^8–15^, whereas the latter is more appropriate for energy harvesting and solar cell design^16–23^. In literature, broadband and narrowband absorbers are generalized terms referred to as any absorber of high and low absorption, whereas a particular class of absorbers with near-unity absorption, working over a large angle of incidence, and showing polarization independency, is termed as superabsorber (or perfect absorber)^24^. In 2008 Landy et al. designed and fabricated a perfect absorber, based on the coupling of electric and magnetic fields to the metamaterial constituents, and facilitated the way for approaching strong absorption of the light^25^. During the last decade, with the progress in nanofabrication technology, many patterns have been proposed and manufactured to enhance the absorption in a wide range of wavelengths^26–29^. Meanwhile, the ability of metallic nanoscale structures to excite surface plasmons resonances (SPRs) and enable a strong field enhancement and confinement in a limited space makes them a promising candidate for elements of high-temperature-based absorbers. These structures can unprecedentedly subside the reflection of the incoming electromagnetic wave and elevate the absorption by trapping the electromagnetic energy in nanoscale patterns.

A plethora of studies, paying attention to perfect absorbers, has been patterned based on noble metals such as silver (Ag) and gold (Au)^30,31^, due to their remarkable plasmonic properties, as well as novel materials such as graphene and $\text {MoS}_2$^32,33^. Perfect absorbers which have been designed for broadband applications, in particular solar cells, can gather a great amount of energy. This leads to high temperatures in the structure which limits its efficiency and functionality. In this regard, due to the inherently low melting points, the mass applications of noble metals are limited by less weak thermal durability. This thermal endurance would be even worse for low dimension materials because the small size effect should be taken into account which leads to the lower melting point^34^. Moreover, some other low-cost alternative metallic materials such as copper (Cu), despite comparatively a higher melting point, their applications are limited due to the narrowband absorption^35^. On this point, the application of aluminum (Al) is limited because of its plasmonic response in the ultraviolet region, the lack of high-temperature durability, and the potential oxidation which makes this material less durable and applicable at high-temperature absorbency^36,37^. As a result, to meet broadband absorption, higher thermal stability, and favorable plasmonic behavior, refractory plasmonic materials, namely the materials of which physical and chemical properties are stable at high temperatures, have been introduced^38^. Extensive research studies have been made to resolve the optical properties of these materials such as titanium (Ti), tungsten (W), chromium (Cr), and molybdenum (Mo). In addition to the refractory metals, multiple kinds of refractory metal compounds can also be used in perfect absorbers fabrication^39^. In contrast to some refractory compounds which are used for their thermal robustness, TiN has been widely used in plasmonic absorbers for ultra-high temperature applications, because of its premium light absorption, remarkable plasmonic properties, thermal robustness, outstanding hardness, and stable physical and chemical properties^38,40,41^. Unlike noble metals in which their plasmonic resonances are limited at shorter wavelengths, TiN can bring remarkable plasmonic properties in both short and long wavelengths. Titanium dioxide ($\text {TiO}_2$) is another compound of Ti. Owing to its high melting point of $1840\,^\circ \text {C}$^29,42^ and the absorption band in the UV region, $\text {TiO}_2$ has been used as a talented candidate for perfect absorber^43,44^.

In the last decade, many researchers have tried to design and manufacture absorbers with high absorption, typically higher than $90\%$, the broad spectrum from UV to IR, especially in visible light and the full spectrum of solar radiation (280–4000 nm)^45^. To design such photonic devices, having a wide absorption bandwidth, polarization independence, and insensitivity to the direction of the incident light are indispensable. A conventional way to achieve a broadband spectrum is to combine different metallic and dielectric materials to overlap the multiple moderate-Q resonances at different wavelengths which effectively spans a broadband spectrum. To meet these criteria many researchers have reported high-absorption and broadband spectra in visible light, infrared, and particularly in the solar radiation spectrum. For instance, a wideband absorber in the range of 0.75–3.25 µm with an average absorption up to 80$\%$^46^, a Ti-based absorber with the absorption of more than 90$\%$ and an average absorption rate over 93.17$\%$ in a wide range of 166.8–1926.6 nm^47^, a Ti/$\text {SiO}_2$ nearly perfect absorber with an average absorption of 97$\%$ over 712 nm extending from 354 nm to 1066 nm^48^, and a metasurface-based perfect absorber with an average absorption around 97.5$\%$ covering the entire visible band have been reported^49^. In addition to the diversity in the designed structures, plenty of the works concentrated on metasurfaces based on multidisks, conical elements, and pyramid-shaped constituents^50–58^. Since the continuous modification of such structures from bottom to top can initiate different resonances, a broadband absorption can be achieved. Furthermore, in recent research studies, some novel photonic structures such as frequency selective rasorber (FSR) with both wide absorption and transmission bands^59,60^, and innovative reflective meta-mirror with an ultra-wideband and high-efficiency characteristics by tuning the phase dispersion and reflection amplitude have been designed to achieve a wideband spectrum^61^.

In spite of the indisputable advantages of the absorbers that have been reported, there are some drawbacks in terms of their design and applications. Some of the proposals are awkward for manufacturing and have many complex elements, the others are narrowband and applicable either in short wavelengths (UV and visible), or long wavelengths (infrared region), while some others despite their relatively broadband spectrum suffer from less absorption. Therefore, designing a broadband perfect absorber covering a wide range of wavelengths with high absorption in the range UV-IR, in particular, the full spectrum of solar radiation is still challenging. In this work, we propose a metasurface-based high-performance absorber in the wide spectrum from near UV to near IR. The unit cell of the proposed metasurface is composed of a truncated cone made of Ti$\text {O}_2$ which is surrounded by alternative conical Ti$\text {O}_2$/TiN rings, set on TiN/Ti$\text {O}_2$ layers. It is shown that our designed structure can absorb a wide spectrum in the range of 0.2–4.5 µm with absorption higher than 90$\%$. The absorption of the structure is dependent on the truncated cone angle. By optimizing the geometrical parameters including the radius of a truncated cone, we propose a structure that can absorb the solar radiation perfectly. In addition, absorption dependence on the polarization and incident angle of the incoming electromagnetic field is studied.

**Figure 1:**
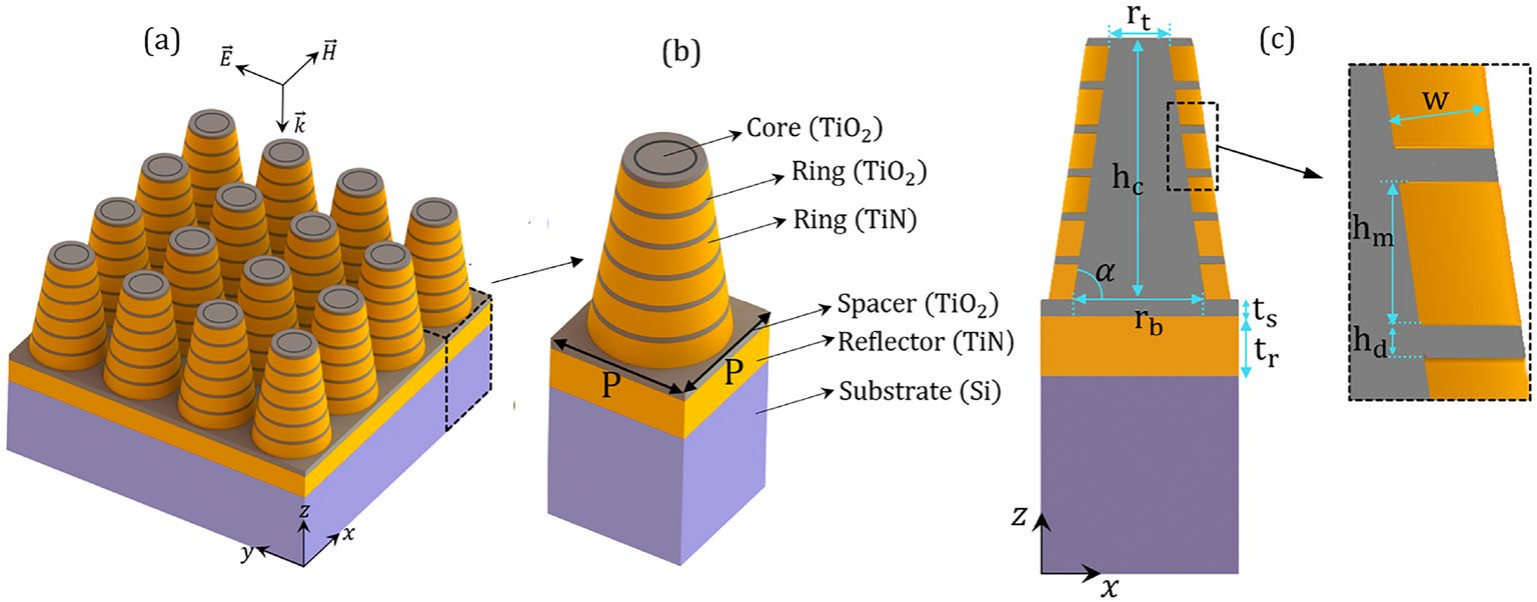
(**a**) Schematic diagram of the proposed metasurface absorber with periodicity $\text{ P }=500~\text {nm}$. (**b**) and (**c**) The unit cell and its *xz*-cross-sectional view. The body of the unit cell is made of a truncated cone-shaped Ti$\text {O}_2$ core of height $\text {h}_\text {c}=660~\text {nm}$ with top and base radii of $\text {r}_\text {t}=101~\text {nm}$ and $\text {r}_\text {b}=200~\text {nm}$ corresponding to the side angle $\alpha =81.47^\circ$. The core is surrounded by conical TiN/Ti$\text {O}_2$ rings of thicknesses $\text{ W }=30~\text {nm}$ and height $\text{ h}_\text {m}=90~\text {nm}$/$\text{ h}_\text {d}=20 ~\text {nm}$ placed on planar Ti$\text {O}_2$/TiN layers of thickness $\text{ t}_\text {s}=40~\text {nm}$/$\text {t}_\text {r}=150~\text {nm}$. The whole array is supported by the Si substrate.

## Results and discussion

**Figure 2:**
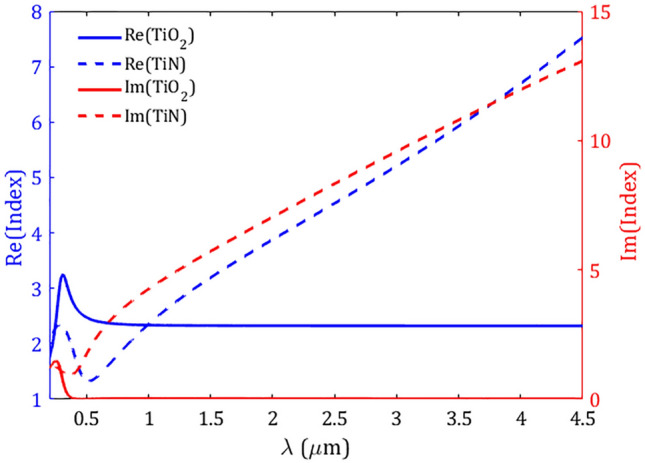
The real (left axis) and imaginary (right axis) of Ti$\text {O}_2$ and TiN refractive indices taken from^62,63^, respectively.

A schematic diagram of the proposed structure is shown in Fig. 1. A metasurface composed of a periodic array of truncated-cone dielectric made of Ti$\text {O}_2$ and covered by alternative N layers of TiN/Ti$\text {O}_2$ rings of the same width W. The whole array is separated from the ground TiN reflector by a Ti$\text {O}_2$ spacer and designed on a silicon (Si) substrate. The periodicity P of the metasurface, defined as the center-to-center distance of two adjacent cones, is the same in both transversal directions (i.e., in the *x* and *y* directions). The unit cell of the proposed metasurface is shown in Fig. 1b. To show the geometrical parameters, in Fig. 1c the *xz*-plane cross-section of the unit cell is shown. Throughout this study the thicknesses of the planar TiN reflective layer of $\text{ t}_\text {r}=150 ~\text {nm}$, and Ti$\text {O}_2$ spacer of $\text {t}_\text {s}=40~\text {nm}$, are kept constant. The bottom and top radii of the truncated-cone core are $\text {r}_\text {b}=200~\text {nm}$ and $\text {r}_\text {t}=101~\text {nm}$, respectively. The multilayer shell is constructed of alternative TiN/Ti$\text {O}_2$ rings of height $\text{ h}_\text {m}=90~\text {nm}$/ $\text{ h}_\text {d}=20 ~\text {nm}$, and the same thicknesses $\text{ W }=30~ \text {nm}$. The height of the truncated Ti$\text {O}_2$ cone is equal to the summation of the heights of entire six TiN/Ti$\text {O}_2$ layers, i.e., $\text {h}_\text {c}=6\times (\text{ h}_\text {m}+\text{ h}_\text {d})=660~\text {nm}$. The side angle of the cone can be calculated easily through $\alpha =\text{ tan}^{-1}(\text{ h}_\text{ c } /(\text {r}_\text {b}-\text {r}_\text {t})) =81.47^\circ$. The dispersive refractive indices of Ti$\text {O}_2$ and TiN are taken from the data of^62,63^, respectively. In Fig. 2 we plotted the fitted FDTD refractive indices of Ti$\text {O}_2$ and TiN in the wavelength range of interest 0.2–4.5 µm.

**Figure 3:**
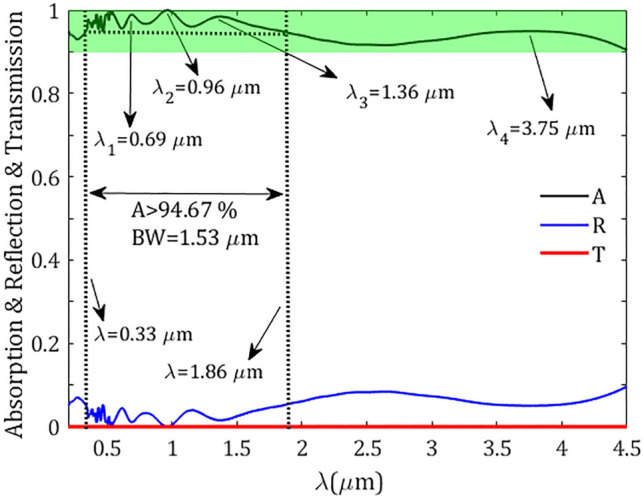
Absorption (A), reflection (R), and transmission (T) spectra of the proposed metasurface absorber in Fig. 1 under a normal incidence. The resonance wavelengths $\lambda _1-\lambda _4$, and absorption bands higher than 90$\%$ (green band) and 94.67$\%$ (dotted lines) in the wavelength range of study starting from 0.2 µm are indicated in the figure.

The absorption, reflection, and transmission spectra of the proposed structure illuminated by a plane wave under normal incidence are shown in Fig. 3. It can be observed that the transmission from the structure is negligible, which is due to the high thickness of the TiN layer, as a result, the incoming energy can be reflected or absorbed by the structure, depending on the interaction of the light and the designed metasurface. Although an amount of the energy is reflected by the pattern, especially at the longest wavelengths, an absorption up to 90$\%$ with an average absorption of 94.71$\%$ in a broad bandwidth of 4.3 µm is revealed. This spectral absorption band covers a wide range of wavelengths spanning in the range of 0.2–4.5 µm, which is exceeding 4-octave bandwidth. In addition, giving consideration to the advantage of high absorption applications in short wavelengths, a high absorption in the visible and its nearby UV and IR lights ranging from 0.33 to 1.86 µm with the absorption higher than 94.67$\%$ and the average absorption 97.25$\%$ can be seen. The broadening of the spectrum can be understood from the formation of a cluster of resonances spread throughout the spectral band of interest. Typically, the size of the resonator defines the resonance characteristics and the possible resonances which can be excited by the incident wave. In our optimized structure due to the existence of a stack of resonators, each resonator can be excited depending on the incident wavelength^56,64–66^. The broadband absorption spectrum is generated by the peak resonances corresponding to the different ingredients of the pattern which can create overall dominant resonances in the spectrum including $\lambda _1=0.69~\upmu \text {m}$, $\lambda _2=0.96~\upmu \text {m}$, $\lambda _3=1.36~\upmu \text {m}$, and $\lambda _4=3.75~\upmu \text {m}$ with high absorption around 98.94$\%$, 99.98$\%$, 98.41$\%$, and 94.95$\%$, respectively.

**Figure 4:**
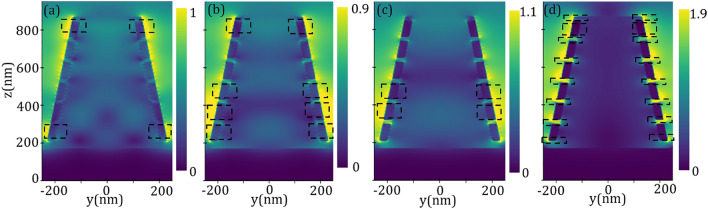
Electric field $\arrowvert \text{ E } \arrowvert$ distributions of the proposed broadband metasurface at *yz*-plane for resonance wavelengths, (**a**) $\lambda _1=0.69~\upmu \text {m}$, (**b**) $\lambda _2=0.96~\upmu \text {m}$, (**c**) $\lambda _3=1.36~\upmu \text {m}$, and (**d**) $\lambda _4=3.75~\upmu \text {m}$. The dashed rectangles localize the enhanced electric fields which are a manifestation for the excitation of the corresponding resonance.

**Figure 5:**
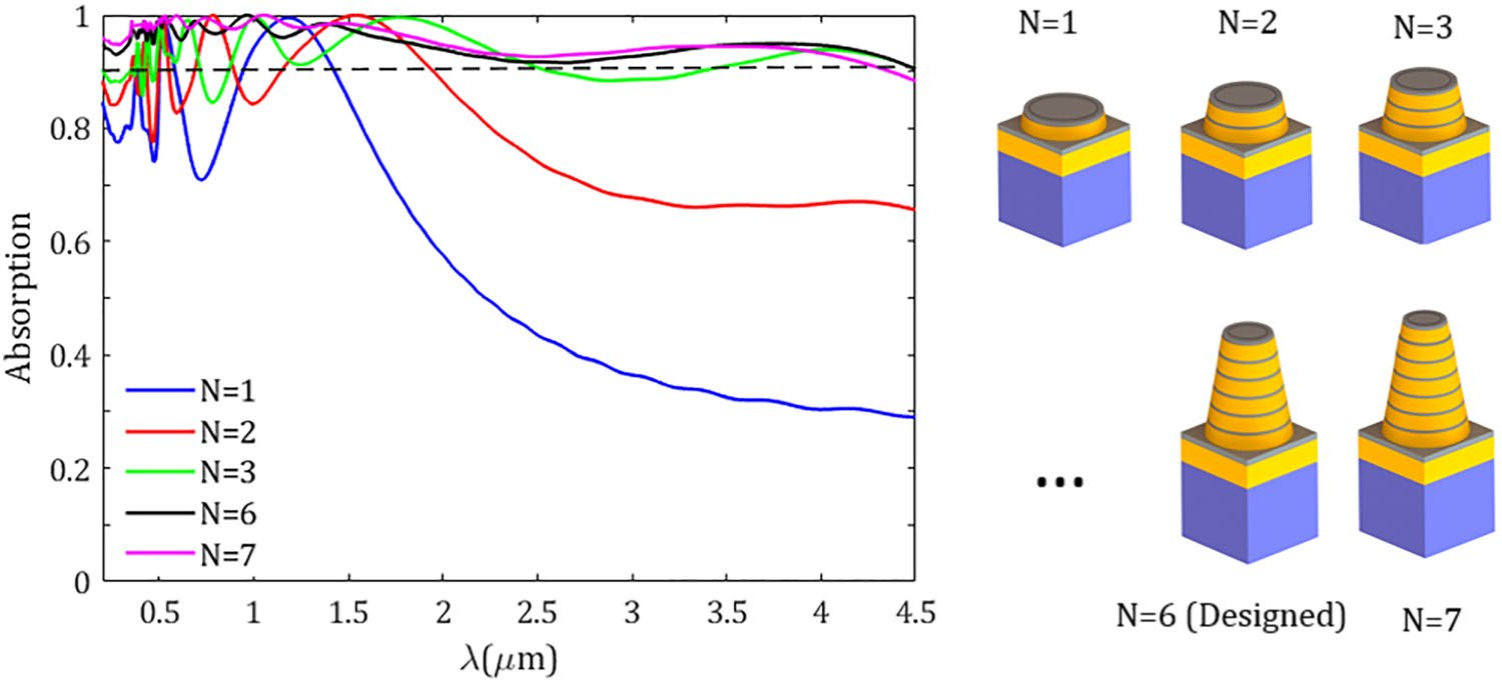
Absorption spectra of the proposed metasurface absorber for a different number N of layers forming the TiN/Ti$\text {O}_2$ rings.

**Figure 6:**
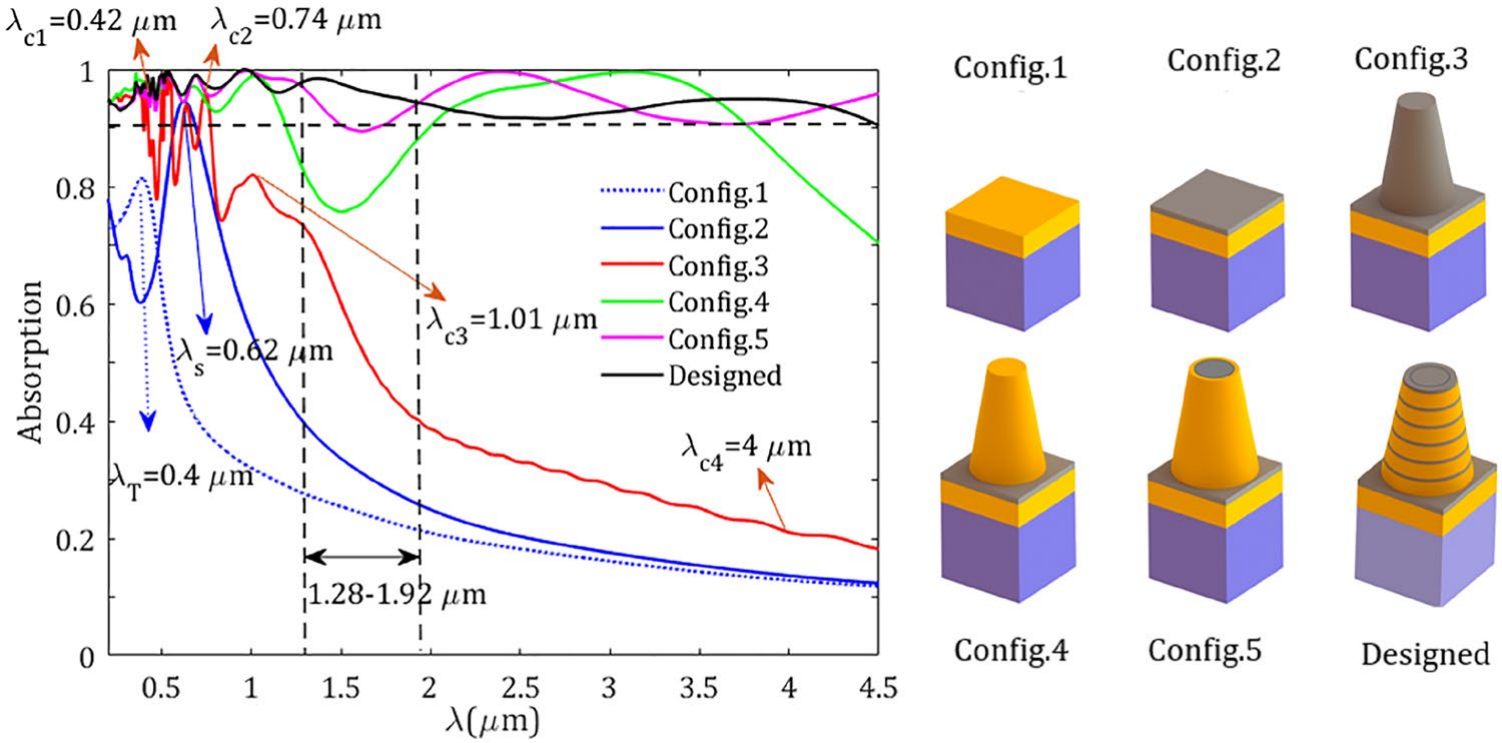
Absorption spectra of different configurations. The resonance wavelengths $\lambda _\text{ T }=0.4~\upmu \text {m}$ for Config.1 and $\lambda _\text{ s }=0.62~\upmu \text {m}$ for Config.2 are indicated in the figure. Here, the resonances $\lambda _{c1}=0.42~\upmu \text {m}$, $\lambda _{c2}=0.74 ~\upmu \text {m}$, $\lambda _{c3}=1.01~\upmu \text {m}$, and low-absorption wavelength $\lambda _{c4}=4~\upmu \text {m}$ of Config.3 are pointed in the figure.

To further get insight into the origin of different resonances and the physical mechanism of the broadband absorption, in Fig. 4 we plotted the magnitudes of the electric field $\arrowvert \text{ E } \arrowvert$ in the *yz*-plane for the above-mentioned resonant maxima taken from Fig. 3. The contours show the normalized electric field to the incident electric field and the dashed rectangles indicate the part of the structure with the most confinement of electric field. As illustrated in Fig. 4a for the resonance of shortest wavelength $\lambda _1$, the electric field is mainly concentrated at the edges of the upper and lowermost TiN rings, which can be attributed to the excitation of localized surface plasmons (LSPs) at the edge of TiN rings. At the resonant peak $\lambda _2$, as illustrated in Fig. 4b, the second TiN ring (from the bottom) has been excited considerably, however, the nearby rings (the bottom and the third rings) and the topmost TiN rings have also been excited to some extent. At resonance $\lambda _3$ the middle TiN rings are strongly responsible for the excitation of plasmonic bound modes (see Fig. 4c). Finally, for the resonance of the longest wavelength $\lambda _4$, an electric field confinement can be seen at the interface of the TiN/Ti$\text {O}_2$ rings and at the side of the upper ring; in this case both the propagating surface plasmons at the interface of TiN/Ti$\text {O}_2$ and LSPs at the side of TiN ring can trigger a strong electric field confinement which leads to a high absorption. It should be noted that the number of stacked layers of the structure has a notable impact on its absorption. In Fig. 5 the absorption spectra of the designed structure for a different number N of layers forming the TiN/Ti$\text {O}_2$ rings were plotted. We kept constant the angle of the cone and other geometrical parameters as in Fig. 3 and varied the height of the cone accordingly, that is $\text {h}_\text {c}=\text{ N }\times (\text{ h}_\text {m}+\text{ h}_\text {d})$. The figure reveals that the absorption of the structure increases significantly as the number of rings increases. However, the absorption enhancement rate is decreased by increasing the number of the rings, i.e., by increasing layer numbers from N=1 to N=2, the absorption enhancement is much higher than the situation when varying N from N=6 to N=7. In addition, the lower rings have more effect on the absorption in longer wavelengths, while the upper rings can considerably affect the absorption enhancement in short wavelengths which is perfectly compatible with our interpretation made in Fig. 4.

**Figure 7:**
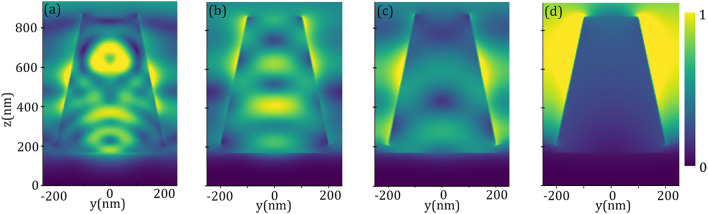
Electric field $\arrowvert \text{ E } \arrowvert$ distributions of the conical Ti$\text {O}_2$ metasurface (Config.3 in Fig. 6) at *yz*-plane for resonance wavelengths, (**a**) $\lambda _{c1}=0.42~\upmu \text {m}$, (**b**) $\lambda _{c2}=0.74~\upmu \text {m}$, (**c**) $\lambda _{c3}=1.01~\upmu \text {m}$, and (**d**) an arbitrary wavelength $\lambda _{c4}=4~\upmu \text {m}$ taken from low-absorption regime.

To see the effect of different elements in the proposed structure, we calculated the absorption for different configurations in Fig. 6. Firstly, we calculated the absorption of a thick TiN layer (150 nm) deposited on Si substrate (Config.1). As illustrated in Fig. 6 (blue-dotted line) in the short wavelengths, there is a narrowband absorption around $\lambda _\text{ T }=0.4~\upmu \text {m}$, while the absorption is decreased as the wavelength increases. Due to the high thickness of TiN, the transmission is negligible, hence the planar structure can substantially reflect the incoming wave. When the TiN layer is covered by Ti$\text {O}_2$ spacer of thickness 20 nm (Config.2), some parts of the energy can be trapped in the spacer which leads to higher absorption in the short wavelengths which is accompanied by a red-shift in the dominant resonance around $\lambda _\text{ s }=0.62~\upmu \text {m}$. For the long wavelengths, similar behavior as a TiN layer can be observed. By introducing a truncated conical Ti$\text {O}_2$ core the absorption is slightly increased, specifically in shorter wavelengths, which can be attributed to the excitation of cone resonances (Config.3). To see this more clearly, in Fig. 7 the magnitudes of electric field are plotted for the resonances $\lambda _{c1}=0.42~\upmu \text {m}$, $\lambda _{c2}=0.74~\upmu \text {m}$, $\lambda _{c3}=1.01~\upmu \text {m}$, and an arbitrary long wavelength in the low-absorption regime $\lambda _{c4}=4~\upmu \text {m}$, where the corresponding absorptions are 94.6$\%$, 95.8$\%$, 81.0$\%$, and 21.0$\%$, respectively . As one can see from Fig. 7a,b, for the resonances $\lambda _{c1}=0.42 ~\upmu \text {m}$ and $\lambda _{c2}=0.74~\upmu \text {m}$ the electric fields are highly confined inside the cone, however for the resonance $\lambda _{c3}=1.01~\upmu \text {m}$, which has lower absorption, the electric field is well localized at the walls of the Ti$\text {O}_2$ resonator. For the long wavelengths such as $\lambda _{c4}=4~\upmu \text {m}$ (Fig. 7d), there is negligible confinement of energy inside the resonator which leads to the low absorption in the spectrum. It should be mentioned that the high value of the imaginary index of Ti$\text {O}_2$ in the short wavelengths can assist in higher absorption in this regime (see Fig. 2 and^43,44^).

Now let us get back to the Config.4 in Fig. 6, where the Ti$\text {O}_2$ core has been replaced by a cone made of TiN. In this case, due to the metallic behavior of TiN and excitation of surface plasmons resonances, the absorption is increased comparatively. When a conical Ti$\text {O}_2$ core is covered by a TiN shell an absorption enhancement can be observed (Config.5). Although, in this case a relatively higher absorption can be observed in short wavelengths, but this structure undergoes a low absorption in the short infrared wavelengths in the approximated range 1.28–1.92 µm which leads to an absorption band with absorption higher than 89.4$\%$ in the whole spectrum. In this case, the broadband absorption can be achieved through the adiabatically change in the size of the rings which are continuously covered the central cone and scaled down from bottom to top of the cone, as a result, the resonances occur at different heights of the TiN shell^56,64–66^. Finally, our optimized structure demonstrates higher absorption in the whole spectrum with a continuous absorption band higher than 90$\%$ (Designed).

**Figure 8:**
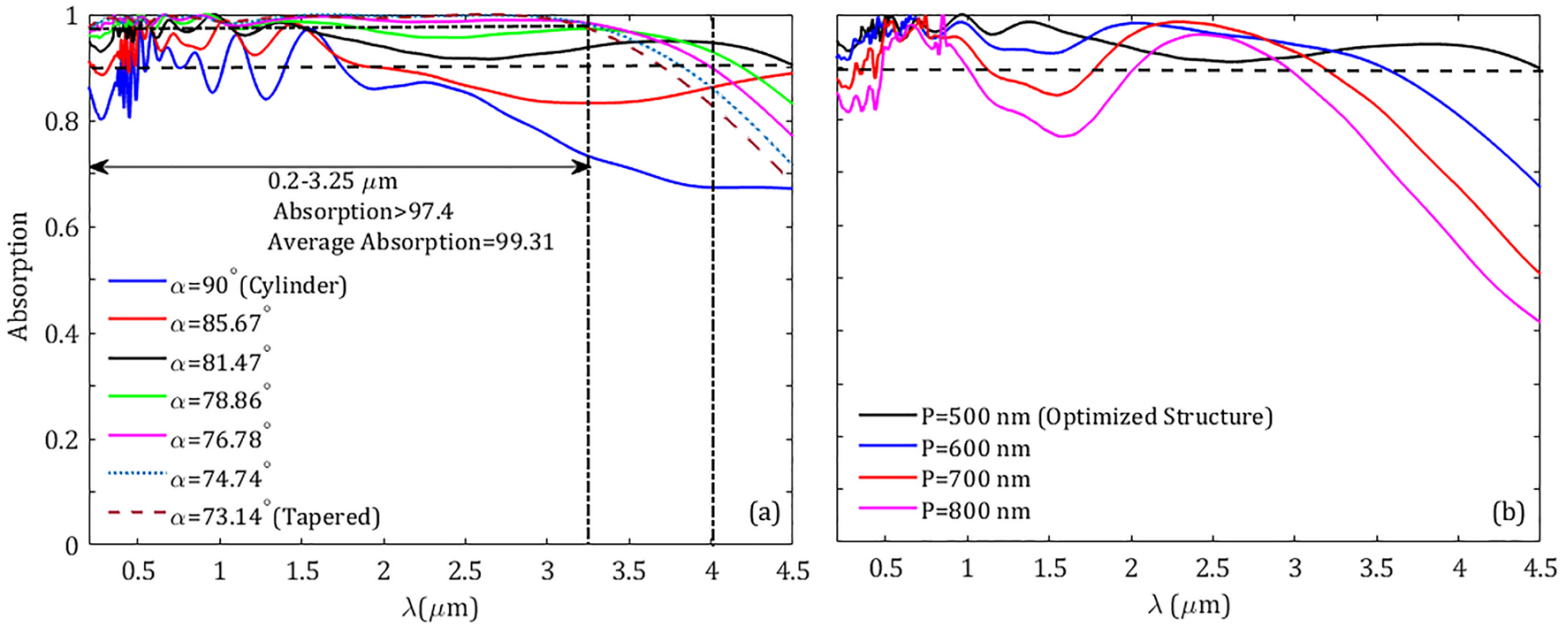
The absorption spectra for the designed metasurface, (**a**) varying the side angle $\alpha$ of the truncated cone, and (**b**) for different values of the periodicity P of arrays. The other parameters are kept constant as Fig. 3.

**Table 1:** Absorption band for absorption >90$\%$ versus different side angles of the cone in the range of 0.2–0.45 µm.

$\alpha (\text {deg})$	Band edges (µm)	Bandwidth (µm)	Average absorption ($\%$)
90	–	–	–
85.67	0.45–1.96	1.51	94.66
81.47	0.2–4.50	4.30	94.71
78.86	0.2–4.19	3.99	96.83
76.78	0.2–4.00	3.80	98.11
74.74	0.2–3.83	3.63	98.61
73.14	0.2–3.70	3.50	98.63

Next, we present how the side angle of the cone can affect the absorption of the proposed structure. In Fig. 8a we plotted the absorption for different side angles of the truncated cone varying from the critical values $\alpha =90^\circ$ (a cylindrical core) to $\alpha =73.14^\circ$ (tapered cone). Additionally, in order to quantitatively compare different schemes, in Table 1 we have tabulated the values of boundaries of the absorption spectral band, the bandwidth, and the corresponding average absorption. It can be observed from the figure, when the elements of the structure are cylindrical-shaped, in the whole spectrum range (0.2–4.5 µm), there is no broadband response with absorption >90$\%$. The absorption of the structure in both short and long wavelengths increases as long as the side angle $\alpha$ decreases, in such a way that spectral bands with absorption higher than 90$\%$ can be created for the values $\alpha =85.67^\circ$ and $\alpha =81.47^\circ$ in the wavelength ranges 0.45–1.96 µm and 0.2–4.50 µm, respectively. The latter case is the optimized structure with the absorption band covering the whole defined spectrum from near-ultraviolet to near-infrared, 4.3 µm wide. By further decreasing the top radius of the cone (i.e., decreasing the side angle of the cone), the absorption in short wavelengths increases while it decreases at the limit of longer wavelengths. From Fig. 8a, one can see setting the side angle $\alpha =76.78^\circ$ an absorption band in the range 0.2–4 µm can be observed which perfectly coincides with the full spectrum of solar radiation whereas the average absorption is significantly as high as 98.11$\%$. By further tapering the cone, the absorption in short wavelengths stands high while in the longer wavelengths it slightly experiences a reduction, where for the tapered angle $\alpha =73.14^\circ$ ($\text{ r}_\text{ t }=0$) a super absorption is created in the wavelength range 0.2–3.25 µm with absorption higher than 97.40$\%$ and dramatically average absorption 99.31$\%$. Such vigorous absorption attributed to our proposal with the broadband response is much more favorable than previously reported approaches for full solar spectrum absorption. To better compare our structure with other absorbers we tabulated the characteristics of some relevant references in Table 2.

**Table 2:** Comparison of our proposed superabsorber with recently-reported relevant absorbers.

References	Band edges (µm)	Bandwidth (µm)	Average absorption ($\%$)
Our proposal ($\alpha =81.47^\circ$)	0.2–4.5	4.3	94.71
Our proposal ($\alpha =76.78^\circ$)	0.2–4	3.8	98.11
Our proposal ($\alpha =73.14^\circ$)	0.2–3.25	3.05	99.31
Ref.^58^	0.25–3.7	3.45	97.5
Ref.^57^	0.3–2.54	2.24	99.17
Ref.^56^	0.1–2.5	2.4	96.11
Ref.^47^	0.1668–1.9266	1.7598	93.17
Ref.^54^	0.2–4	3.8	93
Ref.^53^	0.4–1.5	1.1	99.6
Ref.^48^	0.354–1.066	0.712	97
Ref.^52^	0.4–0.85	0.45	98.1
Ref.^49^	0.4–0.76	0.36	97.50

**Figure 9:**
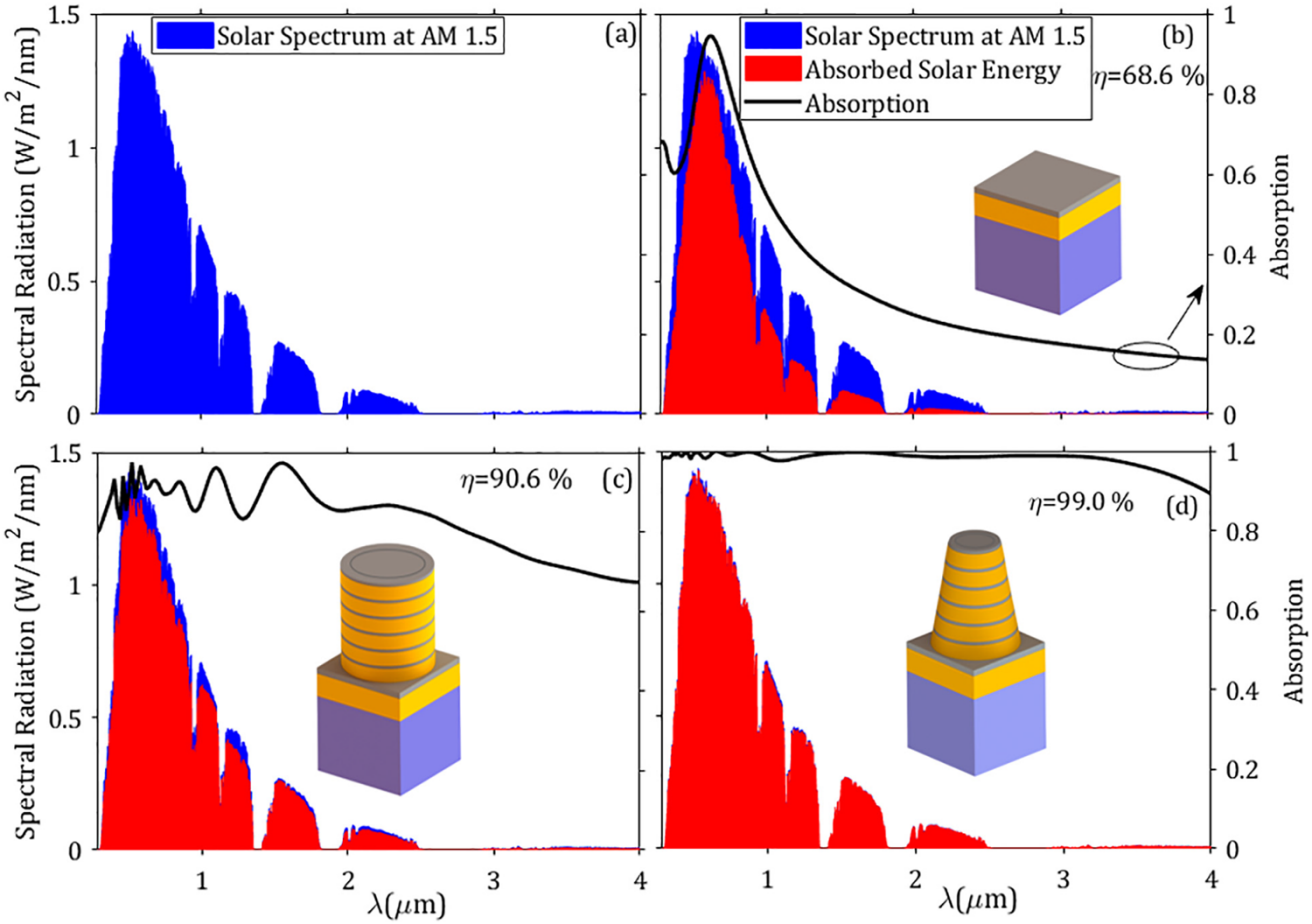
Absorption of the proposed metasurface under a solar spectrum at AM1.5. (**a**) solar radiation at AM1.5. (**b**–**d**) are the absorbed energy of the planar structure, metasurface with cylindrical elements, and metasurface with truncated cones of $\alpha =76.78^\circ$, respectively.

The other parameter that can critically affect the absorption response is the periodicity of the metasurface. Figure 8b shows the absorption spectra of the proposed structure for different values of the periodicity P. The figure reveals that for the values of P greater than the optimized structure (P=500 nm) the absorption of the structure is mostly reduced in the spectral band of interest. This is more evident in the long-wavelength ranges. This is mainly caused by the fact that the effective area of metasurface covered by the periodic array is comparatively reduced and the separation between neighboring cones is increased, which reduces the interaction of a given unit cell with the adjacent unit cells. It is worth noting that the periodicity of the structure along with the geometrical parameters such as the thickness and the height of the metasurface elements have a crucial role in its absorption band edges. The band edges of metasurface absorbers can be tuned to longer wavelengths such as infrared and microwave when the geometrical parameters are chosen appropriately^67–70^.

The absorption level and the bandwidth of the proposed structure can be tuned through the top radius of the truncated cone in the wide range of 0.2–4.5 µm. Since the solar energy is mainly concentrated in the range 0.28–4 µm with more than half of its energy in the UV and visible light, and the rest in the infrared region. Thus, high absorption in this range is of particular interest in solar energy harvesting applications. To better quantify the advantage of the proposed structure as a solar absorber, we introduce the solar absorption efficiency $\eta$ as:

1 $$\begin{aligned} \eta =\frac{\int _{\lambda _{min}}^{\lambda _{max}} \text{ A }(\lambda ) \, \text{ I}_{\text {AM1.5}}(\lambda ) \, d \lambda }{\int _{\lambda _{min}}^{\lambda _{max}} \text{ I}_{\text {AM1.5}}(\lambda ) \, d \lambda }, \end{aligned}$$

where, $\text{ I}_{\text {AM1.5}}(\lambda )$ is the spectral intensity of solar radiation in the US continent taken from the global tilt AM1.5 data, and $\lambda _{min}=0.28~\upmu \text{m}$ and $\lambda _{max}=4~\upmu \text{m}$ are the minimum and maximum of the solar radiation wavelengths at AM1.5^45^.

**Table 3:** Comparison of the absorption efficiency $\eta$ of our proposed solar superabsorber with some reported relevant structures.

References	Structure	$\eta$ (%)
This work	Conical TiN/Ti$\text {O}_2$ rings array	99
Ref.^71^	Cubic W–Al$_2$O$_3$ array	96
Ref.^72^	W/Si$\text {O}_2$ ring-disc array	97
Ref.^73^	Si$\text {O}_2$/Ti$\text {O}_2$/W hyperbolic metamaterials	95
Ref.^74^	TiN/TiNO/Zr$\text {O}_2$/Si$\text {O}_2$ nanofilms	92
Ref.^75^	Triangular Ti-based array	91
Ref.^76^	Mo truncated-cone array	92

Figure 9 shows the solar radiation at AM1.5 and the associated absorption of three different cases illuminated by solar radiation AM1.5. Additionally, the absorption of the respective structure under a plane wave illumination of unit intensity is plotted on the right axis. As can be seen from Fig. 9b, the unstructured planar surface can partially absorb the solar energy, in particular in the visible, and nearby ultraviolet and infrared lights. In this case, using Eq. (1), the absorption efficiency yields $68.6\%$. When cylindrical metasurface elements are introduced in the planar structure, as it is shown in Fig. 9c the solar energy can be absorbed more efficiently ($90.6 \%$). Finally, for the tapered-like cone $\alpha =76.78^\circ$ due to the high absorption in the whole spectrum of interest, the absorption curve is perfectly matched with the solar radiation. As a result, the absorption efficiency rises dramatically up to $99 \%$ which indicates that the proposed structure is a superb optical absorber in light gathering applications such as solar cells and thermal energy collectors when compared with some reported absorption efficiencies in Table 3.

**Figure 10:**
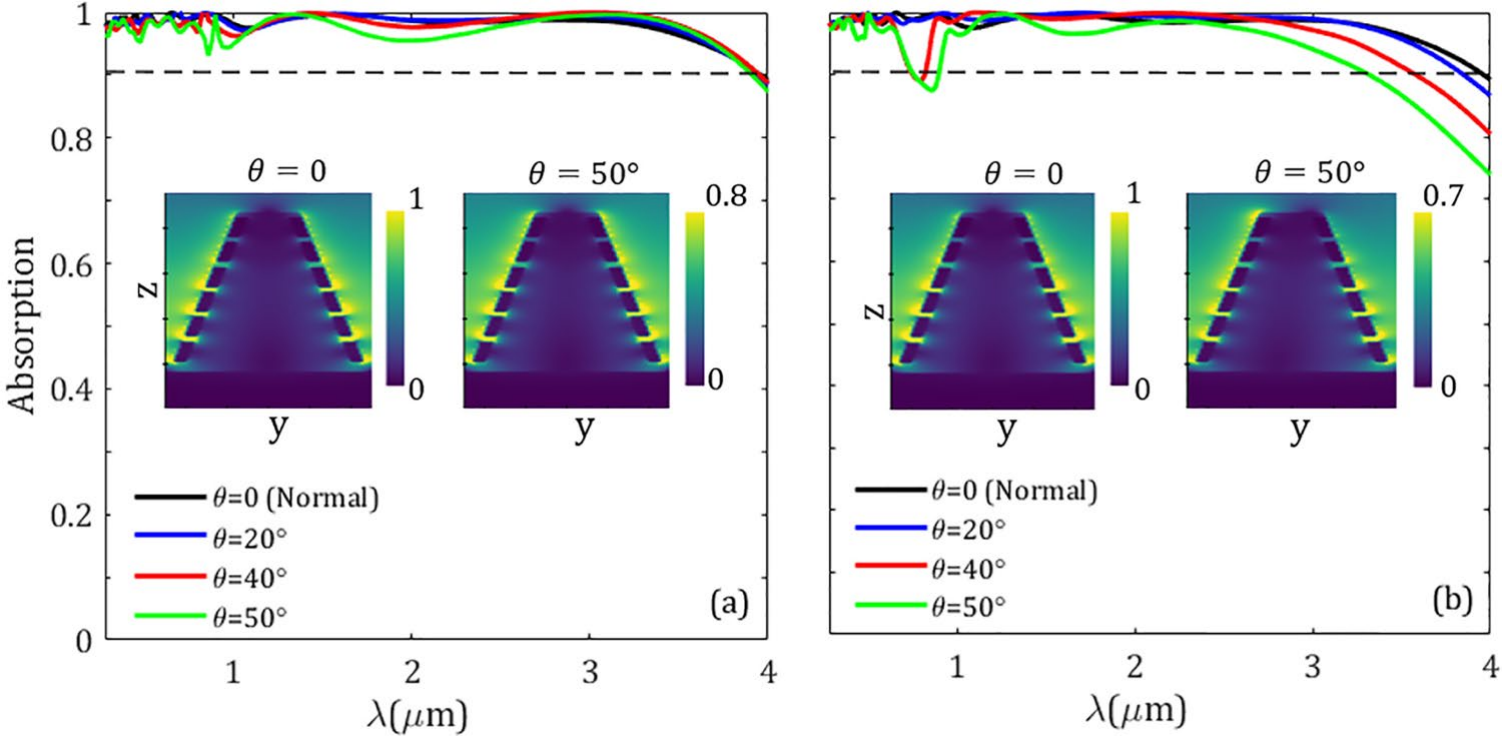
Absorption spectra for different incident angles. (**a**) TE polarization and (**b**) TM polarization. In the inset, the magnitude of the electric field $\arrowvert \text{ E } \arrowvert$ is displayed in the *yz*-plane for normal and oblique incidence at wavelength $\lambda =4~\upmu \text {m}$. Note that the electric field of the launching beam is always in the *yz*-plane for both polarizations.

**Table 4:** Absorption levels and absorption efficiencies for different incident angles of TE and TM polarizations.

$\theta (\deg )$	Minimum Absorption ($\%$)		Average Absorption ($\%$)		Absorption Efficiency $\eta (\%)$	
	TE	TM	TE	TM	TE	TM
0	90	90	98.11	98.11	99	99
20	88.52	86.62	98.14	98.02	98.8	99.13
40	88.73	80.47	98.03	96.42	98.14	97.31
50	87.37	73.96	97.16	93.81	97.46	95.6

In addition to broadband and high-level absorption, an optimal superabsorber should prove polarization independence and wide-angle absorption characteristics. To do so, in Fig. 10 we depicted the absorption spectra for different incident angles $\theta$ (the angle between the wave vector of the incident plane wave and the opposite direction of the *z* axis) for transverse electric/magnetic (TE/TM) polarizations, in which the electric/magnetic field is in the ${\hat{x}}/{\hat{y}}$ direction. Here, we simulate the structure of a metasurface with a conical element of $\alpha =76.78^\circ$ for different angles of incidence. For the sake of comparison, in Table 4 we tabulated the minimum level of absorption, average absorption, and absorption efficiency in the spectral band of interest. It can be observed from Fig. 10 and Table 4 that with the increase of incident angle, the absorption of both polarizations is slightly decreased. For a wide range of incidence angles until $50^\circ$, the given structure can provide high absorption performance within the solar spectrum. We point out that such a dependence of the absorption on the launching angle is more sensitive to the TM polarization. In the inset of Fig. 10 the magnitude of electric field $\arrowvert \text{ E } \arrowvert$ is depicted in the *yz*-plane of normal ($\theta =0$) and oblique ($\theta =50^\circ$) incidence for wavelength $\lambda =4~\upmu \text {m}$. The magnitude of the electric field is dropped by factors 0.8 and 0.7 for TE and TM polarizations, respectively. Since the absorption efficiency over the volume of an optical element is proportional to the intensity $\arrowvert \text{ E } \arrowvert ^2$^77^, thus the absorption of TM polarization is more reduced in comparison to the TE polarization. Moreover, from Table 4 it can be seen that the absorption efficiency $\eta$ is slightly reduced with the increment of the incident angle, in a manner, by changing the incident angle from normal to $50^\circ$ the absorption efficiency is dropped from $\eta =99\%$ to $\eta =97.46\%$ and $95.6\%$ for TE and TM polarizations, respectively.

Finally, in practical terms of fabrication over a large area, various techniques with good optical performance have been applied for the preparation of cone-based metasurfaces by combining temperature-controlled deposition. For instance, by means of a pulsed laser deposition method^78^, and texturing via chemical lithography^79^ or focused-ion-beam (FIB) milling^80^. Particularly FIB technology is potentially useful here since it enables precise tapering by using grayscale bitmaps to control the beam dose during the FIB milling. Alternatively, nanopatterning via colloidal nanosphere lithography^56,81^ have demonstrated high flexibility and tunability, additionally enabling fabrication over a large surface area, thus becoming a technology to be considered here. To conclude, our proposal can benefit from multiphoton polymerization that is a promising technique well suited if increasing the complexity of the building block in the metasurface, demonstrating the highest resolution of all additive manufacturing techniques^66,82,83^.

## Conclusions

In summary, we presented a metasurface superabsorber consisting of an array of truncated conical $\text {TiO}_2$ surrounded by alternating conical TiN/$\text {TiO}_2$ rings. The metasurface elements were placed on a relatively thick layer of TiN which strongly diminishes the transmission of the structure. The numerical calculations demonstrated that the designed structure can absorb a normal-incident electromagnetic wave in the band of 4.3 µm wide with nominal absorption higher than $90\%$, and average absorption $94.71\%$ in the whole spectrum of the study, i.e., 0.2–4.5 µm. The simulated electric field distributions show that the overall broadband high absorption band can be attributed to the excitation of individual modes of each elementary resonator included at different heights of the conical unit cell, and surface plasmons resonances. The absorption of our design can be well modified over a wide range of wavelengths by tailoring the metasurface elements from cylindrical- to tapered-shape through the radius of the tapered side of the conical unit cell. The results indicated that when the unit cell is cylindrical-shaped no considerable absorption can be achieved. By tapering the truncated cone the absorption can be increased significantly so that for the tapered-like cone, an absorption band 3.8 µm wide in the range 0.2–4 µm with absorption higher than 90$\%$ was created. This spectral band, matching the full solar spectrum radiation, has an approximate average absorption of $98.11\%$. In particular, there exists a band in the range 0.2–3.25 µm with absorption higher than 97.40$\%$ and substantially average absorption over 99$\%$. This made our designed metasurface for gathering solar radiation. So that, the proposed structure illuminated by solar radiation at AM1.5 can dramatically absorb more than $99\%$ of solar radiation in its full spectrum (0.28–4 µm). Additionally, our optimal structure has a weak sensitivity to the polarization and incident angle of the incoming electromagnetic wave. By changing the incident angle from normal to $50^\circ$ the absorption efficiency is decreased from $\eta =99\%$ to $\eta =97.46\%$ and $\eta =95.6\%$ for TE and TM polarizations, respectively. As a consequence, the proposed structure can maintain its high absorbance for oblique incidences which can be applied for thermal energy harvesting, solar absorption, and infrared detection.

## Modeling and methodology

Our proposed structure in Fig. 1 is illuminated by a plane wave of unit amplitude propagated in the opposite direction of the *z* axis with linearly-polarized electric $\text{ E}_\text{ y }$ and magnetic $\text{ H}_\text{ x }$ fields in the *y* and *x* directions. For numerical simulations we applied finite-difference time-domain (FDTD) method using Lumerical FDTD package^84^. The simulation was implemented in $0.5~\upmu \text{m} \times 0.5~\upmu \text{m} \times 1.1~\upmu \text{m}$ in a 3D *xyz* box. The mesh size 4 nm in all directions was employed to ensure the numerical convergence. Thanks to the periodicity of the metasurface, we modeled a unit cell of the metasurface and applied proper periodic boundary conditions (for normal incidence) in *xz*- and *yz*-planes and perfectly matched layer (PML) in the *z* direction. To launch a normal plane wave of wavelength ${\lambda }$, we set up a plane source (perpendicular to *z* direction) 80 nm above the top side of the cone with the wavelength in the range of 0.2–4.5 µm. In order to calculate the wavelength-dependent reflection $\text{ R }({\lambda })$ and transmission $\text{ T }({\lambda })$ of the structure, we positioned the frequency-domain field and power monitors in the planes 50 nm above the source and bottom of the TiN reflector, respectively. Due to the thick layer of TiN which is much larger than its penetration depth, the transmission would be substantially negligible, therefore it can be considered to be zero as:

2 $$\begin{aligned} \text{ T }({\lambda })=0. \end{aligned}$$

As a result, absorption $\text{ A }({\lambda })$ directly depends on reflectance, which is calculated by:

3 $$\begin{aligned} \text{ A }({\lambda })={1-\text{ R }(\lambda ).} \end{aligned}$$

## Data Availability

The datasets used and/or analysed during the current study are available from the corresponding author on reasonable request.

## References

[1] Schuller JA (2010). Plasmonics for extreme light concentration and manipulation. Nat. Mater..

[2] Shen Y (2013). Plasmonic gold mushroom arrays with refractive index sensing figures of merit approaching the theoretical limit. Nat. Commun..

[3] Bakker RM (2015). Magnetic and electric hotspots with silicon nanodimers. Nano Lett..

[4] Ghahremani M, Habil MK, Zapata-Rodriguez CJ (2021). Anapole-assisted giant electric field enhancement for surface-enhanced coherent anti-stokes raman spectroscopy. Sci. Rep..

[5] Habil MK, Zapata-Rodríguez CJ, Cuevas M, Entezar SR (2021). Multipolar-sensitive engineering of magnetic dipole spontaneous emission with a dielectric nanoresonator antenna. Sci. Rep..

[6] Kang Q, Li D, Wang W, Guo K, Guo Z (2021). Multiband tunable thermal camouflage compatible with laser camouflage based on GST plasmonic metamaterial. J. Phys. D: Appl. Phys..

[7] Kang Q, Li D, Guo K, Gao J, Guo Z (2021). Tunable thermal camouflage based on GST plasmonic metamaterial. Nanomaterials.

[8] Cheng F, Yang X, Gao J (2014). Enhancing intensity and refractive index sensing capability with infrared plasmonic perfect absorbers. Opt. Lett..

[9] Yong Z, Zhang S, Gong C, He S (2016). Narrow band perfect absorber for maximum localized magnetic and electric field enhancement and sensing applications. Sci. Rep..

[10] Rifat AA, Rahmani M, Xu L, Miroshnichenko AE (2018). Hybrid metasurface based tunable near-perfect absorber and plasmonic sensor. Materials.

[11] Lan G (2020). Narrowband perfect absorber based on dielectric-metal metasurface for surface-enhanced infrared sensing. Appl. Sci..

[12] Cheng Y, Chen F, Luo H (2020). Triple-band perfect light absorber based on hybrid metasurface for sensing application. Nanoscale Res. Lett..

[13] Rakhshani MR (2020). Three-dimensional polarization-insensitive perfect absorber using nanorods array for sensing and imaging. IEEE Sens. J..

[14] Pan M (2020). A narrowband perfect absorber with high Q-factor and its application in sensing in the visible region. Results Phys..

[15] Li Z (2021). Three-band perfect absorber with high refractive index sensing based on an active tunable Dirac semimetal. Phys. Chem. Chem. Phys..

[16] Ma C, Yan J, Huang Y, Wang C, Yang G (2018). The optical duality of tellurium nanoparticles for broadband solar energy harvesting and efficient photothermal conversion. Sci. Adv..

[17] Nagarajan A, Vivek K, Shah M, Achanta VG, Gerini G (2018). A broadband plasmonic metasurface superabsorber at optical frequencies: Analytical design framework and demonstration. Adv. Opt. Mater..

[18] Li J (2020). Broadband solar energy absorber based on monolayer molybdenum disulfide using tungsten elliptical arrays. Mater. Today Energy.

[19] Elsharabasy A, Bakr M, Deen MJ (2020). Wide-angle, wide-band, polarization-insensitive metamaterial absorber for thermal energy harvesting. Sci. Rep..

[20] Lin K-T, Lin H, Yang T, Jia B (2020). Structured graphene metamaterial selective absorbers for high efficiency and omnidirectional solar thermal energy conversion. Nat. Commun..

[21] Qiu Y, Zhang P, Li Q, Zhang Y, Li W (2021). A perfect selective metamaterial absorber for high-temperature solar energy harvesting. Solar Energy.

[22] Piao R, Zhang D (2021). Ultra-broadband perfect absorber based on nanoarray of titanium nitride truncated pyramids for solar energy harvesting. Phys. E: Low-Dimens. Syst. Nanostruct..

[23] Erkmen F, Ramahi OM (2021). A scalable, dual-polarized absorber surface for electromagnetic energy harvesting and wireless power transfer. IEEE Trans. Microw. Theory Tech..

[24] Abdulkarim YI (2022). A review on metamaterial absorbers: Microwave to optical. Front. Phys..

[25] Landy NI, Sajuyigbe S, Mock JJ, Smith DR, Padilla WJ (2008). Perfect metamaterial absorber. Phys. Rev. Lett..

[26] Azad AK (2016). Metasurface broadband solar absorber. Sci. Rep..

[27] Jung J, Park H, Park J, Chang T, Shin J (2020). Broadband metamaterials and metasurfaces: A review from the perspectives of materials and devices. Nanophotonics.

[28] Raad SH, Atlasbaf Z, Zapata-Rodríguez CJ (2020). Broadband absorption using all-graphene grating-coupled nanoparticles on a reflector. Sci. Rep..

[29] Yao Y (2021). Refractory materials and plasmonics based perfect absorbers. Nanotechnology.

[30] Li Z, Butun S, Aydin K (2014). Ultranarrow band absorbers based on surface lattice resonances in nanostructured metal surfaces. ACS Nano.

[31] Li Q (2019). Tunable perfect narrow-band absorber based on a metal-dielectric-metal structure. Coatings.

[32] Zhao Z (2018). Sub-wavelength grating enhanced ultra-narrow graphene perfect absorber. Plasmonics.

[33] Luo X, Zhai X, Wang L, Lin Q (2018). Enhanced dual-band absorption of molybdenum disulfide using a plasmonic perfect absorber. Opt. Express.

[34] Jiang Q, Zhang S, Zhao M (2003). Size-dependent melting point of noble metals. Mater. Chem. Phys..

[35] Park JW (2013). Multi-band metamaterial absorber based on the arrangement of donut-type resonators. Opt. Express.

[36] Knight MW (2014). Aluminum for plasmonics. ACS Nano.

[37] Zhou L (2016). 3D self-assembly of aluminium nanoparticles for plasmon-enhanced solar desalination. Nat. Photon..

[38] Guler U, Boltasseva A, Shalaev VM (2014). Refractory plasmonics. Science.

[39] Guler U (2013). Local heating with lithographically fabricated plasmonic titanium nitride nanoparticles. Nano Lett..

[40] Li W (2014). Refractory plasmonics with titanium nitride: Broadband metamaterial absorber. Adv. Mater..

[41] Qin F (2020). Ultra-broadband and wide-angle perfect solar absorber based on TiN nanodisk and Ti thin film structure. Solar Energy Mater. Solar Cells.

[42] Gülşen G, Inci MN (2002). Thermal optical properties of TiO$$_2$$ films. Opt. Mater..

[43] Dahl M, Liu Y, Yin Y (2014). Composite titanium dioxide nanomaterials. Chem. Rev..

[44] Chen X, Liu L, Huang F (2015). Black titanium dioxide (TiO$$_2$$) nanomaterials. Chem. Soc. Rev..

[45] Air Mass 1.5 Spectra, American society for testing and materials (ASTM). https://www.nrel.gov/grid/solar-resource/spectra-am1.5.html.

[46] Liu X, Fu G, Liu M, Zhan X, Liu Z (2019). Titanium nanoholes meta-surface for ultra-broadband infrared absorption. Results Phys..

[47] Yu P (2020). Ultra-wideband solar absorber based on refractory titanium metal. Renew. Energy.

[48] Lei L, Li S, Huang H, Tao K, Xu P (2018). Ultra-broadband absorber from visible to near-infrared using plasmonic metamaterial. Opt. Express.

[49] Qian Q, Sun T, Yan Y, Wang C (2017). Large-area wide-incident-angle metasurface perfect absorber in total visible band based on coupled Mie resonances. Adv. Opt. Mater..

[50] Li Q, Gao J, Yang H, Liu H (2016). A super meta-cone absorber for near-infrared wavelengths. Plasmonics.

[51] Lu Y, Lal A (2010). High-efficiency ordered silicon nano-conical-frustum array solar cells by self-powered parallel electron lithography. Nano Lett..

[52] Huo D (2017). Broadband perfect absorber with monolayer MoS$$_2$$ and hexagonal titanium nitride nano-disk array. Nanoscale Res. Lett..

[53] Huo D (2018). Broadband perfect absorber based on TiN-nanocone metasurface. Nanomaterials.

[54] Mehrabi S, Rezaei MH, Zarifkar A (2019). Ultra-broadband solar absorber based on multi-layer TiN/TiO$$_2$$ structure with near-unity absorption. JOSA B.

[55] Dang PT (2020). Efficient broadband truncated-pyramid-based metamaterial absorber in the visible and near-infrared regions. Crystals.

[56] Guo Z (2021). Near-perfect broadband metamaterial absorbers of truncated nanocones using colloidal lithography. Opt. Mater..

[57] Liu H, Xie M, Ai Q, Yu Z (2021). Ultra-broadband selective absorber for near-perfect harvesting of solar energy. J. Quant. Spectrosc. Radiat. Transf..

[58] Li X (2022). Full spectrum ultra-wideband absorber with stacked round hole disks. Optik.

[59] Shen Z, Kou N, Yu S, Ding Z, Zhang Z (2020). Miniaturized frequency selective rasorber based on meander-lines loaded lumped resistors and a coupled resonator spatial filter. Prog. Electromagn. Res. M.

[60] Zhang Y-X, Ban Y-L, Sim C-Y-D (2021). Ultra-wideband RCS reduction of circular polarization slot antenna array based on polarization conversion structures and frequency-selective rasorber. Prog. Electromagn. Res. M.

[61] Cai T (2020). Ultrawideband chromatic aberration-free meta-mirrors. Adv. Photon..

[62] Siefke T (2016). Materials pushing the application limits of wire grid polarizers further into the deep ultraviolet spectral range. Adv. Opt. Mater..

[63] Palik, E. D. *Handbook of Optical Constants of Solids* Vol. 3 (Academic press, 1998).

[64] Cui Y (2012). Ultrabroadband light absorption by a sawtooth anisotropic metamaterial slab. Nano Lett..

[65] Liang Q (2013). Numerical study of the meta-nanopyramid array as efficient solar energy absorber. Opt. Mater. Express.

[66] Khodasevych IE, Wang L, Mitchell A, Rosengarten G (2015). Micro-and nanostructured surfaces for selective solar absorption. Adv. Opt. Mater..

[67] Ding F, Cui Y, Ge X, Jin Y, He S (2012). Ultra-broadband microwave metamaterial absorber. Appl. Phys. Lett..

[68] Ye L (2019). Ultra-wideband terahertz absorption using dielectric circular truncated cones. IEEE Photon. J..

[69] Nochian P, Atlasbaf Z (2020). A novel single layer ultra-wideband metamaterial absorber. Prog. Electromagn. Res. Lett..

[70] Malik S (2021). Design and analysis of polarization-insensitive broadband microwave absorber for perfect absorption. Prog. Electromagn. Res. M.

[71] Qin F (2022). Broadband solar absorbers with excellent thermal radiation efficiency based on W-Al$$_2$$O$$_3$$ stack of cubes. Int. J. Therm. Sci..

[72] Yi Z (2020). Broadband polarization-insensitive and wide-angle solar energy absorber based on tungsten ring-disc array. Nanoscale.

[73] Jiang X, Wang T, Zhong Q, Yan R, Huang X (2020). Ultrabroadband light absorption based on photonic topological transitions in hyperbolic metamaterials. Opt. Express.

[74] Li Y (2019). Scalable all-ceramic nanofilms as highly efficient and thermally stable selective solar absorbers. Nano Energy.

[75] Li Y (2018). Efficient, scalable, and high-temperature selective solar absorbers based on hybrid-strategy plasmonic metamaterials. Solar RRL.

[76] Wang J, Chen Z, Li D (2010). Simulation of two-dimensional Mo photonic crystal surface for high-temperature solar-selective absorber. Phys. Status Solidi A.

[77] Akimov YA, Koh W (2010). Resonant and nonresonant plasmonic nanoparticle enhancement for thin-film silicon solar cells. Nanotechnology.

[78] Sugavaneshwar RP (2017). Fabrication of highly metallic tin films by pulsed laser deposition method for plasmonic applications. ACS Photon..

[79] Liu G (2018). Large-area, low-cost, ultra-broadband, infrared perfect absorbers by coupled plasmonic-photonic micro-cavities. Solar Energy Mater. Solar Cells.

[80] Ding F (2014). Ultrabroadband strong light absorption based on thin multilayered metamaterials. Laser Photon. Rev..

[81] Wang J (2017). Large-scale broadband absorber based on metallic tungsten nanocone structure. Appl. Phys. Lett..

[82] Su V-C, Chu CH, Sun G, Tsai DP (2018). Advances in optical metasurfaces: Fabrication and applications. Opt. Express.

[83] Askari M (2020). Additive manufacturing of metamaterials: A review. Addit. Manuf..

[84] Lumerical Inc. Lumerical FDTD. https://www.lumerical.com/products/fdtd/.

